# Novel MPPT algorithm based on honey bees foraging characteristics for solar power generation systems

**DOI:** 10.1016/j.heliyon.2024.e27491

**Published:** 2024-03-07

**Authors:** Wei-Jen Chen, Shoeb-Azam Farooqui, Hwa-Dong Liu, Shan-Xun Lai, Ping-Jui Lin

**Affiliations:** a Undergraduate Program of Vehicle and Energy Engineering, National Taiwan Normal University, Taipei, 106, Taiwan; b Department of Electrical Engineering, National Taiwan University of Science and Technology, Taipei, 106, Taiwan

**Keywords:** Honey bee dancing, Solar power system, Maximum power point tracking, Uniform irradiance condition, Partial shaded condition

## Abstract

This study proposes a novel honey bee dancing (HBD) maximum power point tracking (MPPT) algorithm inspired by the foraging behavior of honey bees. When a bee finds nectar, it returns to the honeycomb and dances to inform others about the location of the nectar. Other bees then fly towards the location and gather the nectar. The proposed HBD algorithm uses five bees searching for the nectar who communicate with each other about the location and the quantity of nectar by dancing. Finally, the five bees found the location of the most nectar which is represented by the maximum power point. The proposed HBD algorithm applies to uniform irradiance condition (UIC) and partial shaded condition (PSC). It is then compared with the PV panel output power and load relationship (OPLR) algorithm and perturb and observe (P&O) algorithm. Experimental verification has been performed under both the UIC and PSC. At the UIC's irradiance level of 200 W/m^2^, the PV module's output power for the proposed HBD algorithm, OPLR algorithm, and P&O algorithm are 120 W, 118 W, and 94.5 W, respectively, with efficiencies of 99 %, 97 %, and 78 %. Additionally, under the PSC with an irradiance level of 600 W/m^2^, the PV module's output power for the proposed HBD algorithm, OPLR algorithm, and P&O algorithm are 218 W, 189.2 W, and 74.8 W, respectively, with efficiencies of 99 %, 86 %, and 34 %, and convergence times of 4.7 ms, 6.5 ms, and 6 ms, respectively. It is evident from the results that the solar MPPT performance of the proposed HBD algorithm is better than the OPLR algorithm and P&O algorithm. This method ingeniously combines the foraging behavior of bees with solar power generation to produce the maximum natural power. This approach does not require the development of photovoltaic (PV) panel specification data, complex calculations, and additional temperature meters and heliographs. It is highly efficient and has significant economic benefits.

## 1 Introduction

The 27th Conference of the Parties (COP27) [1] aspires to achieve net zero emissions in the middle of this century, reduce 50% of carbon emissions by 2030, and control the global average temperature rise within 1.5 °C. Therefore, governments across the world strive to reduce carbon emission, eliminate fossil fuels progressively, and transition towards 100% renewable energy. Renewable energy is energy harnessed from natural sources and offers the notable advantage of being inexhaustible. It includes wind power, tidal energy, geothermal energy, hydraulic power, and solar energy [[2], [3], [4], [5]]. Solar photovoltaic (PV) system is one of the most ecologically beneficial and globally accessible form of electricity generation [3].

Many studies on applications of PV have been presented in the literature. Some of the related studies are described as follows [[6], [7], [8], [9], [10], [11], [12], [13], [14]]. Ref [6] discusses the application of the self-acculturative damped filter in the parallel connection system of PV power and grid so that when the grid is unstable, PV power can be used to maintain high power quality at the load end. Reference [7] introduces a PV system integrated with an enhanced multilayer second-order generalized integrator. This approach allows the PV system not only to supply power to its designated load but also to feed surplus power back into the grid. Ref [8] studies the application of PV and wind power generation in electric vehicle battery charging. The system combines a multilevel reoriented generalized integrator to facilitate electric vehicle charging, and redirecting the extra power to the AC bus. Ref [9] discusses that the PV power generation system uses a voltage source converter combined with an interweaved generalized integrator, so that the PV power generation system has good performance and provides high-quality power for a three-phase AC Bus. Ref [10] studies the PV grid-connected system combined with the multifarious harmonic elimination method, which eliminates the third, fifth, seventh, and ninth power harmonics, and this system outputs high-quality power to the load end. Ref [11] discusses the combination of model predictive control strategy and modified-dual second-order generalized integrator in PV power generation system so that the system can control the renewable energy and provide stable power to AC loads. Ref [12] proposes a combination of independent solar energy and maximum power point tracking (MPPT) based on the concept of the centerline of a circle to eliminate the vibration problem of the traditional MPPT at the maximum power point (MPP). This method can be close to the MPP to improve system performance. Ref [13] studies the solar inverter combined with anti-windup feedback second-order generalized integrator. This method can stably operate the PV power generation system to maintain stable power output and has low power harmonics so that the load end can obtain a good power quality. Ref [14] introduces the PV power generation system that combines adaptive control technology with a grid, and the system provides battery charging for electric vehicles. This technology enables rapid and secure charging and resulting in a substantial boost in system performance.

These PV generation systems need a solar MPPT controller to capture the MPP under different climatic conditions, and significantly enhancing the system output and efficiency. Therefore, a plethora of MPPT algorithm has been developed and studied by many researchers. Saxena et al. developed a Perturb & Observe (P&O) algorithm based on the circle center-line (CCL) concept, which can effectively predict the perturbation step length, allowing the operating point to quickly approach the MPP and stably operate at the MPP. However, this method still needs to be further applied under partial shaded conditions (PSC) to verify the effectiveness of this algorithm in different environmental conditions [12]. Soeidat et al. discussed the classic P&O algorithm. This method is simple, easy to implement, and is economically effective, but inefficient in a shaded environment [15]. Sahu et al. used the Maclaurin series expansion (MSE) MPPT algorithm to estimate the PV panel *I*_*pv*_-*V*_*pv*_ characteristic curve. They estimated the MPP positions in uniform irradiance condition (UIC) and PSC. However, their method requires PV panel parameter data [16]. Jagadeesan et al. developed a two-stage power regulation (TSPR) MPPT algorithm, which can be adapted to the environment where the sunshine changes quickly and can capture the MPP. However, this method is inapplicable to PSC [17]. Khan et al. presented an artificial neural (ANN) MPPT algorithm, which can capture the MPP effectively and accurately. However, some data are required to train the system, which makes the system complicated and demand substantial processing time [18]. Martin et al. proposed an image processing (IP) MPPT control strategy. A camera monitors the PV panel. The PV panel shaded area is determined for analysis, and then the power electronic converter is controlled to achieve the MPP and improving the system output power in PSC effectively [19]. Lu et al. developed a PV panel output power and load relationship (OPLR) MPPT algorithm, which needs a lot of PV panel characteristics and loads data to analyze the MPP position and finally capture the MPP. This method applies to UIC and PSC. However, massive data require estimation, which leads to system load [20]. Choi et al. developed a two-dimensional (2-D) MPPT based on the hill climbing algorithm. This control strategy uses three steps to capture MPP and applies to UIC [21]. Lu et al. discussed a new MPPT that uses an extended state observer (ESO). This method uses the extended state and proportional integral search MPP [22]. Wang et al. proposed an MPPT which is based on negative feedback control (NFC). This MPPT utilizes the relationship between PV panel output voltage and operating frequency and then captures MPP [23]. Yang et al. developed the salp swarm optimization (SSO) based MPPT to enhance the system's ability to capture MPP under both UIC and PSC. This approach synergizes with a solar power system that incorporates an integrated thermoelectric generator and harnessing excess heat to generate additional electricity [24]. Naseem et al. discussed the use of fuzzy control (FC) to improve the system performance by analyzing the MPP based on the relationship between the slope of PV module's output voltage and output power. This method is simple and easy to implement [25]. Kishore et al. hybridized the teaching algorithm and Bee Colony (BC) MPPT algorithm which aptly called as TBC. This method improves the oscillation problem generated by the MPPT operation and can capture MPP under both UIC and PSC [26]. Husain et al. proposed the fast adjustable duty (FAD) MPPT algorithm, which has a simple structure and can quickly find the MPP. However, more experiments are still needed to verify the effectiveness of MPPT under PSC [27]. Husain et al. discussed DC-link capacitor droop (DCLCD) control. This method involves the monitoring of DC-link capacitor of the power converter. When the voltage of the DC-link capacitor drops, the system cannot provide enough power for the load. Then, DCLCD initiates the MPPT to capture MPP. However, this method suffers with slow response [28]. Arjun et al. introduced an iterative analysis method (IAM) for estimating MPPT technology based on the PV module mathematical model. This method primarily estimates the PV *P*_*pv*_-*V*_*pv*_ characteristic curves of each PV module under PSC through the mathematical model, and then estimates the MPP. This method can quickly capture the MPP. However, it requires the collection of PV module specifications data beforehand [29]. Angadi et al. developed an MPPT technique that optimizes circuit system parameters (OCSP) for the P&O algorithm, including the selection of the optimal capacitor for the power converter, digital signal sampling time, and frequency, which facilitates faster convergence to the MPP in MPPT. However, this algorithm does not further discuss the poor performance of P&O under PSC [30]. Husain et al. introduced an MPPT technique for transient analysis and perturbation parameter selection (TAPPS), which further optimizes the step length of perturbation through small signal model analysis during MPPT. This is beneficial for mitigating oscillation issues at the actuating point and allowing the actuating point to quickly approach the MPP. However, the performance of this method under PSC remains to be evaluated [31]. Castaño et al. discussed an artificial bee colony (ABC) MPPT algorithm combined with PI control and analyzed the optimal working voltage of the PV *P*_*pv*_-*V*_*pv*_ characteristic curve. They validated the performance of the proposed ABC algorithm through a hardware-in-the-loop system. However, they noted that more empirical validation of its performance under PSC is still needed [32].

The comparison of fifteen solar MPPT control strategies have been performed and are illustrated in Table 1. It is obvious from the table that the proposed HBD algorithm is better than CCL, MSE, TSPR, ANN, IP, OPLR, 2-D, ESO, NFC, SSO, TBC, IAM, and ABC algorithms in terms of complexity. Additionally, the proposed HBD algorithm does not need parameters and thus simplifying the overall system configuration. Lastly, the proposed HBD algorithm demonstrates an excellent performance under both the UIC and PSC. The performance of the proposed HBD algorithm is better than CCL, P&O, TSPR, 2-D, ESO, NFC, FC, FAD, DCLCD, OCSP, TAPPS, and ABC algorithms.

**Table 1:** Comparison of several solar MPPT control strategies.

Control strategy	Complexity	Parameter	Efficiency under UIC	Efficiency under PSC
CCL [12]	Medium	Unnecessary	High	Medium
P&O [15]	Low	Unnecessary	Medium	Low
MSE [16]	Medium	Necessary	High	High
TSPR [17]	Medium	Unnecessary	High	Medium
ANN [18]	Medium	Necessary	High	High
IP [19]	High	Necessary	High	High
OPLR [20]	Medium	Necessary	High	High
2-D [21]	Medium	Necessary	High	Medium
ESO [22]	Medium	Necessary	High	Medium
NFC [23]	Medium	Necessary	High	Medium
SSO [24]	Medium	Necessary	High	High
FC [25]	Low	Necessary	High	Medium
TBC [26]	Medium	Necessary	High	High
FAD [27]	Low	Necessary	High	Medium
DCLCD [28]	Low	Unnecessary	Medium	Medium
IAM [29]	Medium	Necessary	High	High
OCSP [30]	Low	Necessary	High	Low
TAPPS [31]	Low	Unnecessary	High	Medium
ABC [32]	Medium	Unnecessary	High	Medium
HBD	Low	Unnecessary	High	High

A novel honey bee dancing (HBD) MPPT algorithm has been introduced in this article. This control strategy is inspired from the remarkable behavior of bees. When a bee finds nectar and returns to the honeycomb, it dances to let other bees know the location of the nectar [33]. Using this intricate natural behavior, a unique MPPT control strategy has been developed in this study. The proposed HBD MPPT control strategy is based on five bees looking for nectar. They collect the information of nectar individually, and communicate about the nectar locations by dancing. MPP in this study represents the location of most nectar. The five bees analyze the power points on the solar *P*_*pv*_*-V*_*pv*_ characteristic curve. Finally, the five bees find the MPP and capture the MPP together. The effectiveness of the proposed HBD algorithm was rigorously assessed and compared with the OPLR algorithm and P&O algorithms under the UIC and PSC [29]. The efficiency of the proposed HBD algorithm is 99 % under both the UIC and PSC, and outperform the OPLR algorithm and P&O algorithm. This method is free from the need of extensive data of PV panel specification, complex calculations, and additional temperature meters and heliographs. It is highly efficient and has high economic benefits. This method combines nectar-gathering behavior of bees with the solar power generation to obtain the most natural energy.

## 2 Solar photovoltaic power system

**Fig. 1:**
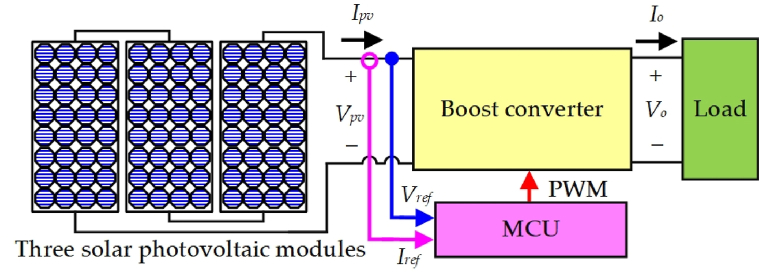
Schematic diagram of three series-connected PV modules connected with boost converter and controller.

Table 2 provides the specification of a single PV module and presenting the key values such that the open-circuit voltage was 36.3 V, the short-circuit current was 7.84 A, the MPP voltage was 29 V, the MPP current was 7.35 A, and the MPP power was 213 W. Fig. 1 is the schematic diagram of three PV modules in combination with a boost converter and controller [34,35]. Initially, the three PV modules were connected in series which results in an MPP of 639 W, an MPP voltage of 87 V, and an MPP current of 7.35 A. Subsequently, the three PV modules were connected to the boost converter and microcontroller unit (MCU). Third, the output voltage signals (*V*_*ref*_), and current signals (*I*_*ref*_) from the three solar PV modules were transmitted to the MCU. Finally, the MCU uses pulse width modulation (PWM) to capture the MPP through the MPPT algorithm.

**Table 2:** Single PV module specification.

Parameter	Value
Open-circuit voltage *V*_*oc*_	36.3 V
Short-circuit current *I*_*sc*_	7.84 A
MPP voltage *V*_*MPP*_	29 V
MPP current *I*_*MPP*_	7.35 A
MP power *P*_*MPP*_	213W

## 3 The proposed MPPT control strategy

**Fig. 2:**
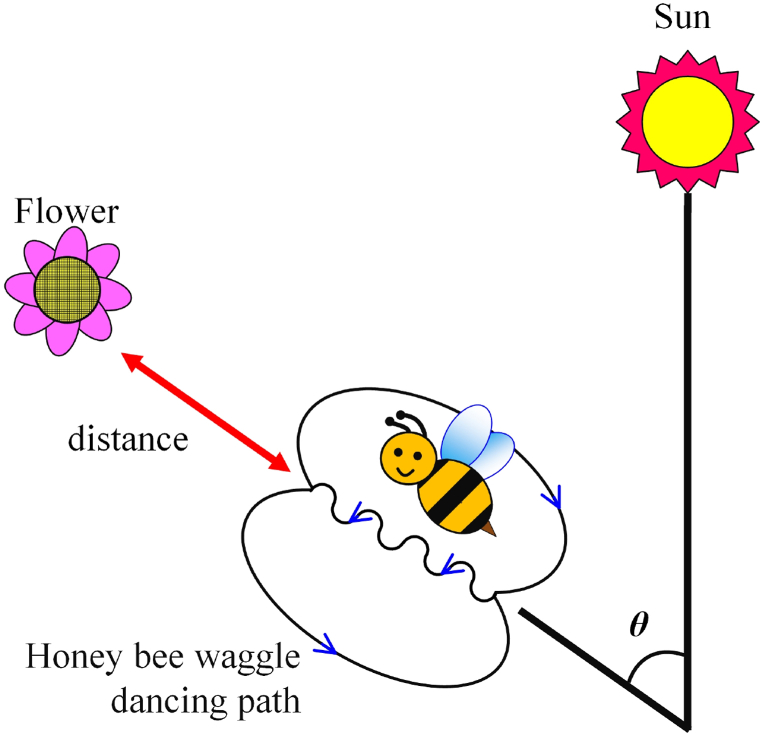
Position diagram of bee, sun, and flower.

**Fig. 3:**
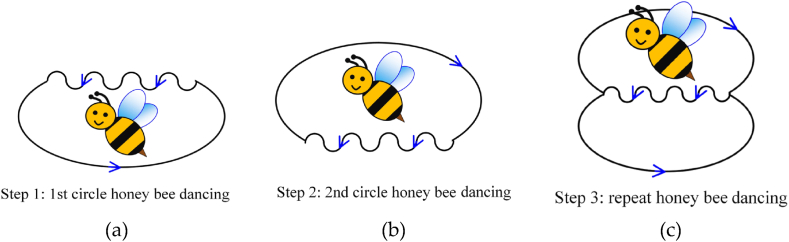
Schematic diagram of 8-shaped dancing sequence. (a) step 1: 1st circle honey bee dancing, (b) step 2: 2nd circle honey bee dancing, and (c) step 3: repeat honey bee dancing.

In terms of bee dancing phenomenon [36], when a bee finds the location of the nectar and returns to the honeycomb, it communicates the nectar location with other bees by dancing. First of all, the dancing bee adjusts its dancing angle (*θ*) in accordance with the position of sun, and aligning its head with the direction of the flowers where the nectar is located (as depicted in Fig. 2). Furthermore, the bee performs a dance following an 8-shaped path (as illustrated in Fig. 3). The bee's 8-shaped dancing sequence is described below. In step 1, the bee wobbles and finishes the first circle (as Fig. 3(a)). In step 2, the bee wobbles for the second time and completes the second circle (as Fig. 3(b)). In step 3, the bee repeats the 8-shaped dance pattern (as Fig. 3(c)). Finally, the bee follows the waggle motion; the waggling of 75 ms means the distance to the flowers (location of nectar) is 100 m. The bee guides other bees to the location of nectar by dancing. This method combines nectar-gathering behavior of bees with solar power generation to obtain the most natural energy.

A novel solar MPPT algorithm has been developed in this study which is inspired from the dancing behavior of a bee to communicate with other bees about the location of nectar. This algorithm is termed as the honey bee dancing (HBD) MPPT control strategy. The waggle seconds of bee dancing represents the variation (△*D*_*Bn*_). The angle (*θ*) in Fig. 2 represents the direction. It can be defined as the positive or negative value of duty cycle *D*_*Bn*_ variation (△*D*_*Bn*_) of the microcontroller unit (MCU) output. The duty cycle (*D*_*Bn*_) of MPPT can be expressed as follows:

(1) $$D_{Bn}=D_{Bn-1}+{\Delta D}_{Bn}$$

wherein *D*_*Bn*_ is the current duty cycle, and *D*_*Bn-1*_ is the past duty cycle. *D*_*B1*_ to *D*_*B5*_ are the positions of the respective bees (*B*_*1*_ through *B*_*5*_) corresponding to the associated duty cycle. ie. *D*_*B1*_ is the position of the first bee (*B*_*1*_) corresponding to the duty cycle. Likewise, *D*_*B2*_, *D*_*B3*_, *D*_*B4*_ and *D*_*B5*_ are the positions of second bee (*B*_*2*_), third bee (*B*_*3*_), fourth bee (*B*_*4*_) and fifth bee (*B*_*5*_) respectively corresponding to their duty cycles.

Many MPPT literatures [30,31] discuss the upper and lower limits of duty cycle and their impact on system stability. To accommodate the comprehensive analysis of the *P*_*pv*_-*V*_*pv*_ curve of the PV module and facilitating system determination of whether the PV module operates in UIC or PSC, a duty cycle range of 0.05–0.95 is set. Consequently, the proposed HBD algorithm allows for a wide variation range in *V*_*pv*_ while maintaining the capability to thoroughly analyze the MPP under both UIC and PSC and ensuring system stability.

**Fig. 4:**
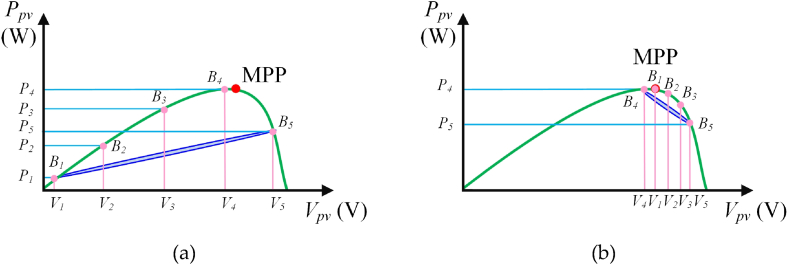
*P*_*pv*_-*V*_*pv*_ characteristic curve of the PV module under UIC using the HBD algorithm: (a) 1st step of the HBD algorithm and (b) 2nd step of the HBD algorithm.

Fig. 4 illustrates the *P*_*pv*_-*V*_*pv*_ curve of PV module under UIC using the HBD algorithm. Fig. 4(a) shows the 1st step of the HBD algorithm on the *P*_*pv*_*-V*_*pv*_ curve under UIC. Five duty cycles which are 0.05, 0.25, 0.5, 0.75, and 0.95, are generated by using the proposed HBD algorithm and are exported through MCU. These duty cycles represent the current positions of the five bees (respectively as *B*_*1*_∼*B*_*5*_). The proposed HBD algorithm estimates the positions of five bees and thus locate five power points (*P*_*1*_∼*P*_*5*_). This curve is approximated to a parabolic curve identifying it as the *P*_*pv*_*-V*_*pv*_ characteristic of UIC. During this time, the five bees operate in the blue oval area enabling the proposed HBD algorithm to traversed two peak power points (*P*_*4*_ and *P*_*5*_). These points (*P*_*4*_ and *P*_*5*_) correspond to the duty cycle (*D*_*B4*_ and *D*_*B5*_) of two bee positions (*B*_*4*_ and *B*_*5*_). The two duty cycles are defined as *D*_*1st*_ and *D*_*2nd*_ respectively. *D*_*1st*_ is smaller than *D*_*2nd*_. The absolute error value (*D*_*m*_) between *D*_*1st*_ and *D*_*2nd*_ are expressed as follows.

(2) $$D_{m}=|D_{1st}-D_{2nd}|$$

(3) $${\Delta D}_{Bn}=\frac{D_{m}}{4}$$

Eq (3) relates the variation in the duty cycle (△*D*_*Bn*_) at MCU output.

In the next step, the equations of the duty cycle (*D*_*Bren*_) corresponding to the positions of the three bees are expressed as follows.

(4) $$D_{Bren}=D_{1st}+ren{\cdot \Delta D}_{Bn}$$

Here, *ren* represents the three bees excluding the bees (*B*_*4*_ and *B*_*5*_) at positions *D*_*1st*_ and *D*_*2nd*_, and the three bees are mentioned as 1, 2, and 3, respectively. Therefore, the proposed HBD algorithm performs iterative computations through Eqs. (1), (2), (3), (4) to capture the MPP (as shown in Fig. 4 (b)). Fig. 4(b) shows the 2nd step of the HBD algorithm on the *P*_*pv*_*-V*_*pv*_ characteristic curve under UIC. During this period, the five bees operate in the blue oval area and this blue oval area is less than that in Fig. 4(a). At this time the Bee (*B*_*1*_) has caught the MPP. Thereby, the proposed HBD algorithm can operate stably in MPP. If the five bees can't catch the MPP in the second calculation, the proposed HBD algorithm performs the iterative computations through Eqs. (1), (2), (3), (4) to capture the MPP.

**Fig. 5:**
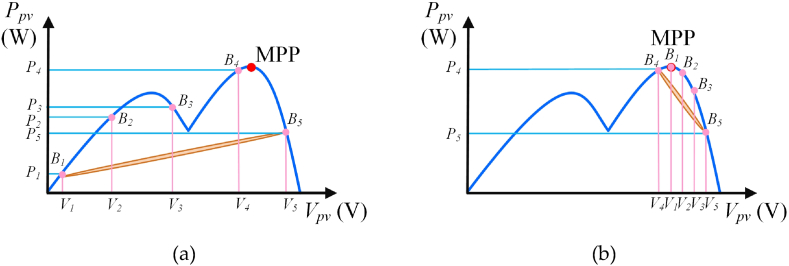
*P*_*pv*_-*V*_*pv*_ characteristic curve of the PV module under PSC using the HBD algorithm: (a) 1st step of the HBD algorithm and (b) 2nd step of the HBD algorithm.

Fig. 5 shows *P*_*pv*_-*V*_*pv*_ curve of PV module under PSC using the HBD algorithm. Fig. 5(a) shows the 1st step of the HBD algorithm on the *P*_*pv*_*-V*_*pv*_ curve under PSC. Five duty cycles which are 0.05, 0.25, 0.5, 0.75, and 0.95, are generated by using the proposed HBD algorithm and are exported through MCU. The five duty cycles represent the current positions of five bees (*B*_*1*_∼*B*_*5*_). The proposed HBD algorithm estimates the positions of five bees and thus locate five power points (*P*_*1*_∼*P*_*5*_). During this time the five bees operate in the orange oval area. According to analysis, this characteristic curve has two peak power points, which are identified as the *P*_*pv*_*-V*_*pv*_ characteristic curve under PSC. The proposed HBD algorithm obtained the positions of the two bees (*B*_*3*_ and *B*_*5*_) on the left and right sides of the bee (*B*_*4*_) at the maximum peak power point (*P*_*4*_), with an MPP in the interval between *B*_*3*_ and *B*_*5*_. Therefore, the proposed HBD algorithm performed iterative computations through Eqs. (1), (2), (3), (4) to capture the MPP.

Fig. 5(b) shows the 2nd step of the HBD algorithm on the *P*_*pv*_*-V*_*pv*_ characteristic curve under PSC. During this period, the five bees operate in the orange oval area and this orange oval area is less than that in Fig. 5(a). At this time the Bee (*B*_*1*_) has caught the MPP. Thereby, the proposed HBD algorithm can operate stably in MPP. If the five bees can't catch the MPP in second calculation, the proposed HBD algorithm performs iterative computations through Eqs. (1), (2), (3), (4) to capture the MPP. Additionally, the researchers can adjust the number of bees according to the actual PV module scale and specification to capture MPP in different environments.

**Fig. 6:**
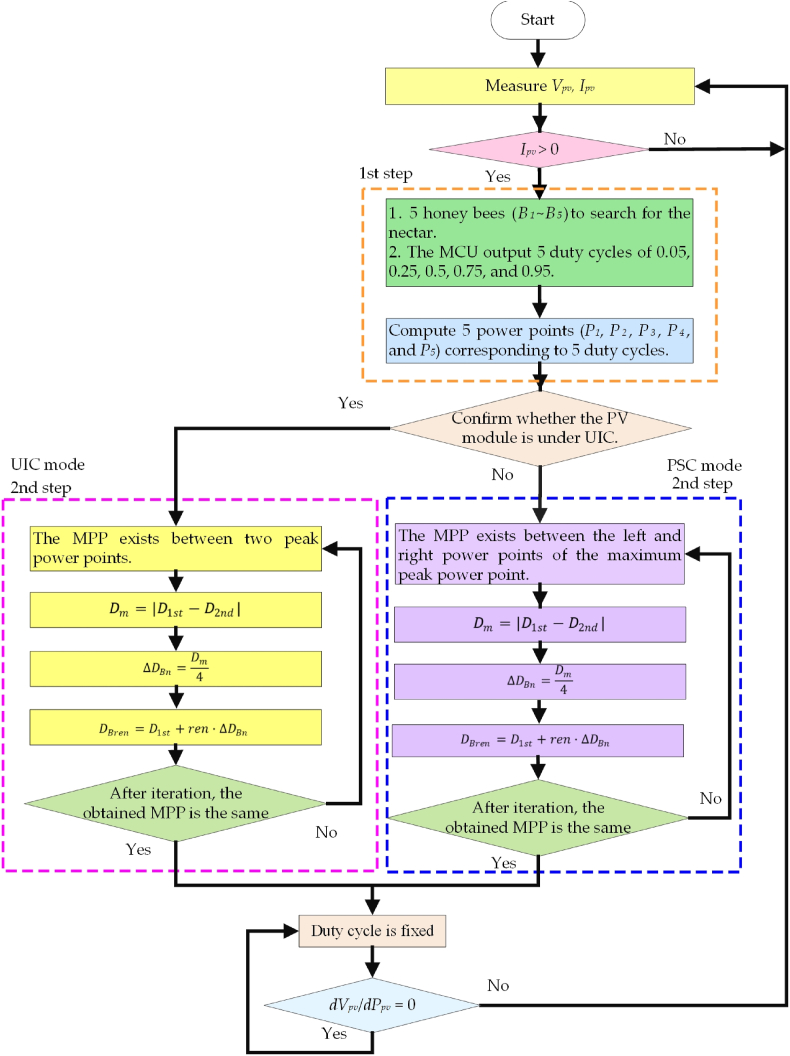
Flowchart of the proposed HBD MPPT control strategy.

Fig. 6 shows the flowchart of the proposed HBD MPPT control strategy. First, the proposed algorithm measured the *V*_*pv*_ and *I*_*pv*_ and verified that *I*_*pv*_ had a current output, and then the proposed algorithm worked. Second, the MCU exported the duty cycles of five bees (*B*_*1*_∼ *B*_*5*_), which were 0.05, 0.25, 0.5, 0.75, and 0.95. The five bees fed back *P*_*pv*_ individually, and then the *P*_*pv*_*-V*_*pv*_ characteristic curve of PV module was analyzed to confirm whether the present PV module was in UIC or PSC. Third, when the PV module was operating under UIC, the proposed HBD algorithm identified the MPP as being situated between two peak power points (as depicted in Fig. 4). To capture this MPP, the algorithm performed iterative computations based on Eqs. (1), (2), (3), (4). Notably, the algorithm converged to the same MPP after two iterations, resulting in a fixed duty cycle. Fourth, when the PV module was operating under PSC, the proposed HBD algorithm discerned the presence of the MPP between the left and right power points of the maximum power point (as illustrated in Fig. 5). The MPP was captured by iterative computations of Eqs. (1), (2), (3), (4). Similar to the UIC case, the algorithm converged to the same MPP after two iterations, resulting in a fixed duty cycle. Ultimately, the proposed HBD algorithm attained the optimal duty cycle and continuously monitored the *dV*_*pv*_/*dP*_*pv*_. The duty cycle was fixed when *dV*_*pv*_/*dP*_*pv*_ = 0. On the contrary, the proposed HBD algorithm performed MPPT again when *dV*_*pv*_/*dP*_*pv*_ ≠ 0.

**Fig. 7:**
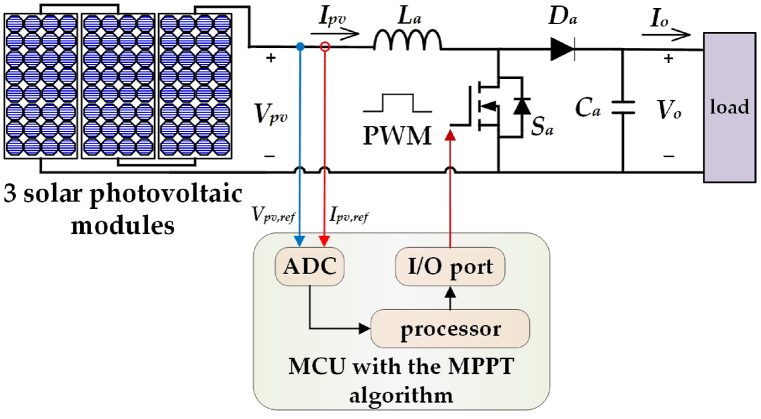
Diagram of the proposed solar power system combined with the MPPT algorithm.

Fig. 7 is the representation of the proposed solar power system combined with the MPPT algorithm. First, three PV modules were connected in series to the boost converter input end with boost converter inductor *L*_*a*_ = 800 μH and capacitor *C*_*a*_ = 330 μF. Second, the boost converter output end was connected to the load. Third, the MCU received the PV module's output voltage and current signals. The voltage and current signals were calculated by the MPPT algorithm. Wherein MCU was Microchip, P/N:18F452. Lastly, the MCU exported 45 kHz PWM to drive the boost converter power MOSFET *S*_*a*_ to capture the MPP.

## 4 Experiments result

**Fig. 8:**
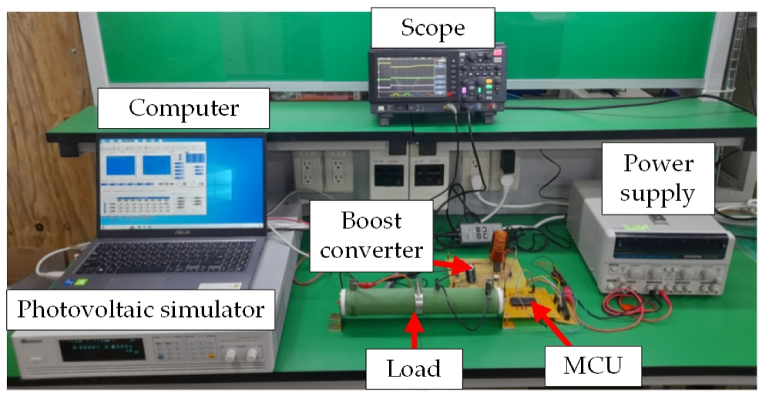
Hardware prototype of the PV system in the laboratory.

Fig. 8 displays the PV system prototype developed in the laboratory. The photovoltaic simulator was Chroma P/N: 62020H. The computer was ASUS, P/N: X515EP-0151G, the scope was Keysight, P/N: DSOX1204A, and the power supply was Gwinstek, P/N: GPS4303. This experiment compared the proposed HBD algorithm, OPLR algorithm, and P&O algorithm in UIC and PSC. Finally, the experimental results verified that the proposed HBD algorithm had better efficiency than the OPLR algorithm and P&O algorithm (as shown in Table 3, Table 4).

**Table 3:** Efficiency comparison of certain algorithm under UIC.

Algorithm	Efficiency	
	Irradiance level = 850 W/m^2^	Irradiance level = 200 W/m^2^
OPLR	99 %	97 %
P&O	99 %	78 %
HBD	99 %	99 %

**Table 4:** Efficiency comparison of HBD, OPLR, and P&O algorithms under PSC.

Algorithm	Efficiency	
	Irradiance level = 600 W/m^2^	Irradiance level = 100 W/m^2^
OPLR	86 %	65 %
P&O	34 %	47 %
HBD	99 %	99 %

### 4.1 Experimental results of UIC

**Fig. 9:**
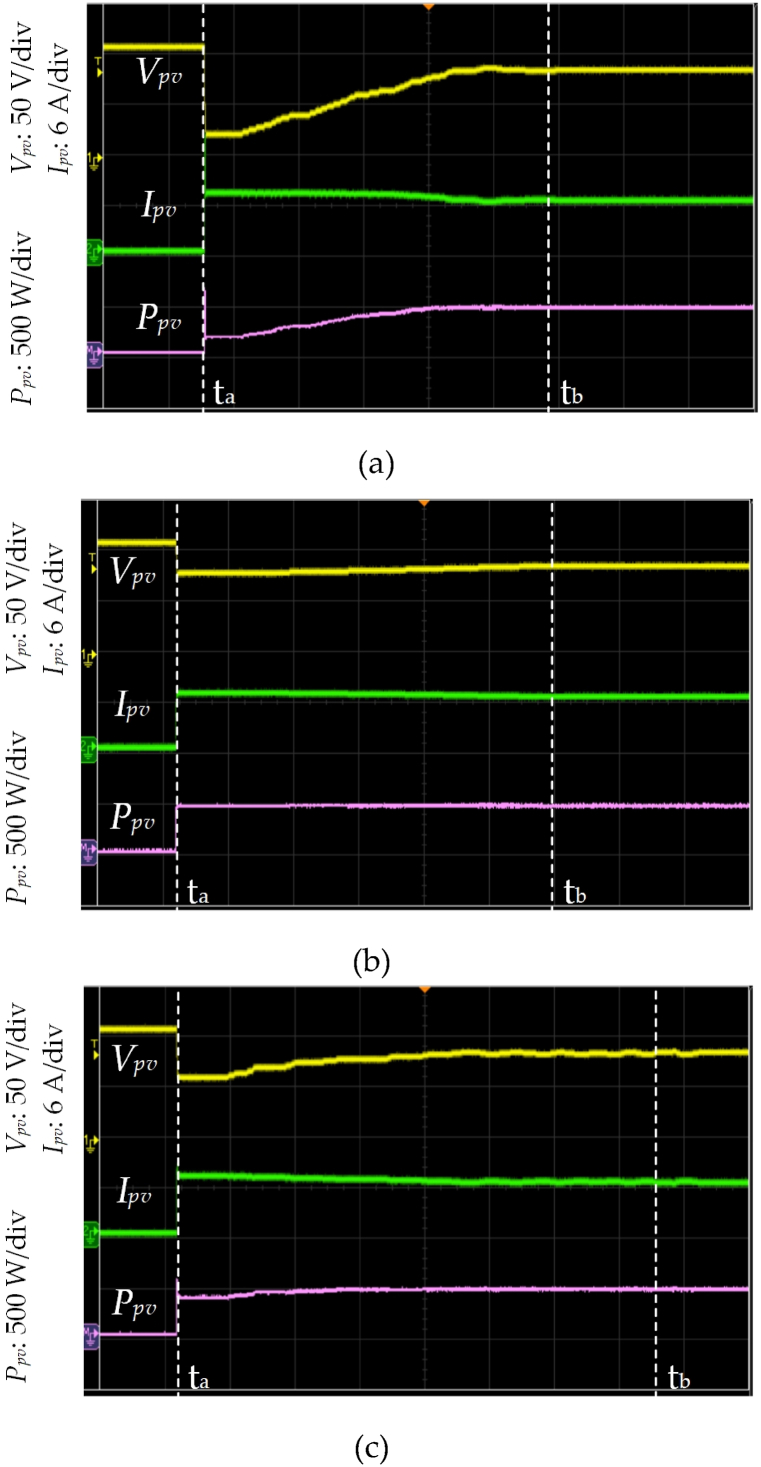
The waveforms of *V*_*pv*_, *I*_*pv*_, and *P*_*pv*_ under UIC with irradiance level = 850 W/m^2^ and temperature = 25 °C by using different control strategies (a) HBD, (b) OPLR, and (c) P&O algorithms (Horizontal axis: 1 ms/div).

**Fig. 10:**
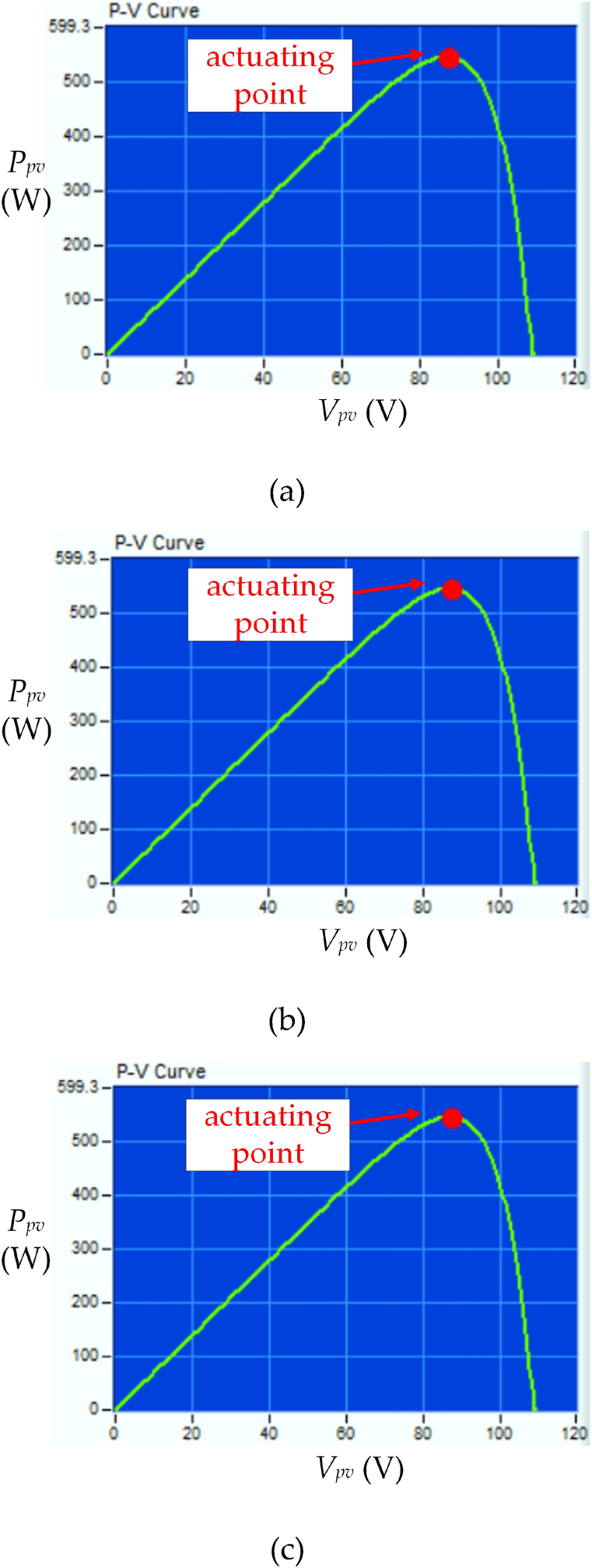
*P*_*pv*_−*V*_*pv*_ characteristic curve of three PV modules in series under UIC at irradiance level = 850 W/m^2^ and temperature = 25 °C by using different control strategies (a) HBD, (b) OPLR, and (c) P&O algorithms.

Fig. 9 shows the waveforms of *V*_*pv*_, *I*_*pv*_, and *P*_*pv*_ under UIC with irradiance level = 850 W/m^2^ and temperature = 25 °C by using different control strategies. Fig. 9 (a) displays the proposed HBD algorithm. The MPPT begins at time t_a_ when *I*_*pv*_ > 0 is detected. The *P*_*pv*_−*V*_*pv*_ curve is analyzed using five honey bees and these bees correspond to duty cycles of 0.05, 0.25, 0.5, 0.75, and 0.95 and resulting in respective *P*_*pv*_ of 50 W, 190 W, 380 W, 520 W, and 290 W. The three PV modules in series operating under UIC are further verified using the proposed algorithm. Subsequently, two MPPs at 380 W and 520 W are obtained. Duty cycles *D*_1*st*_ = 0.5, *D*_*B1*_ = 0.56, *D*_*B2*_ = 0.62, *D*_*B3*_ = 0.68, and *D*_2*nd*_ = 0.75 are calculated using Eqs. (1), (2), (3), (4) (as shown in Fig. 4, Fig. 6). *P*_*pv*_ of 380 W, 410 W, 520 W, 540 W, and 520 W are obtained corresponding to *D*_1*st*_, *D*_*B1*_, *D*_*B2*_, *D*_*B3*_, and *D*_2*nd*_ respectively. The proposed HBD algorithm uses iterative computation to capture the MPP at time t_b_ and achieving the MPPT time of 5.1 ms. The actuating point using the proposed HBD algorithm is *P*_*MPP*_ = 540 W, an efficiency of 99 %, and an MPP duty cycle of 0.62 (as shown in Fig. 10(a)). The proposed HBD algorithm can accommodate a large *V*_*pv*_ variation range while accurately identifying the MPP position.

Fig. 9 (b) shows the OPLR algorithm and the MPPT was initiated at time t_a_. This algorithm successfully captures the MPP at time t_b_ and achieving an MPPT time of 5.8 ms. It operates in a relatively smooth manner to capture the MPP which results in an actuating point at *P*_*MPP*_ = 540 W (as shown in Fig. 10(b)). Fig. 9 (c) shows the P&O algorithm and the MPPT was started at time t_a_. However, this algorithm encounters challenges as it captures the MPP at time t_b_ and resulting in a longer MPPT time of 7.2 ms. Additionally, the *V*_*MPP*_ keeps oscillating. The actuating point for P&O algorithm is determined to be *P*_*MPP*_ = 540 W (as shown in Fig. 10(c)).

Fig. 10 shows the *P*_*pv*_−*V*_*pv*_ characteristic curve of three PV modules in series under UIC at irradiance level = 850 W/m^2^ and temperature = 25 °C using different control strategies. Fig. 10 (a) displays the *P*_*pv*_−*V*_*pv*_ curve using the proposed HBD algorithm. The actuating point using the proposed HBD algorithm is *P*_*MPP*_ = 540 W, an efficiency of 99 % (as Fig. 9(a)). Fig. 10(b) and (c) show that the actuating points using the OPLR and P&O algorithms are achieved at *P*_*MPP*_ = 540 W and the efficiency of 99 % (as Fig. 9(b) and (c)).

**Fig. 11:**
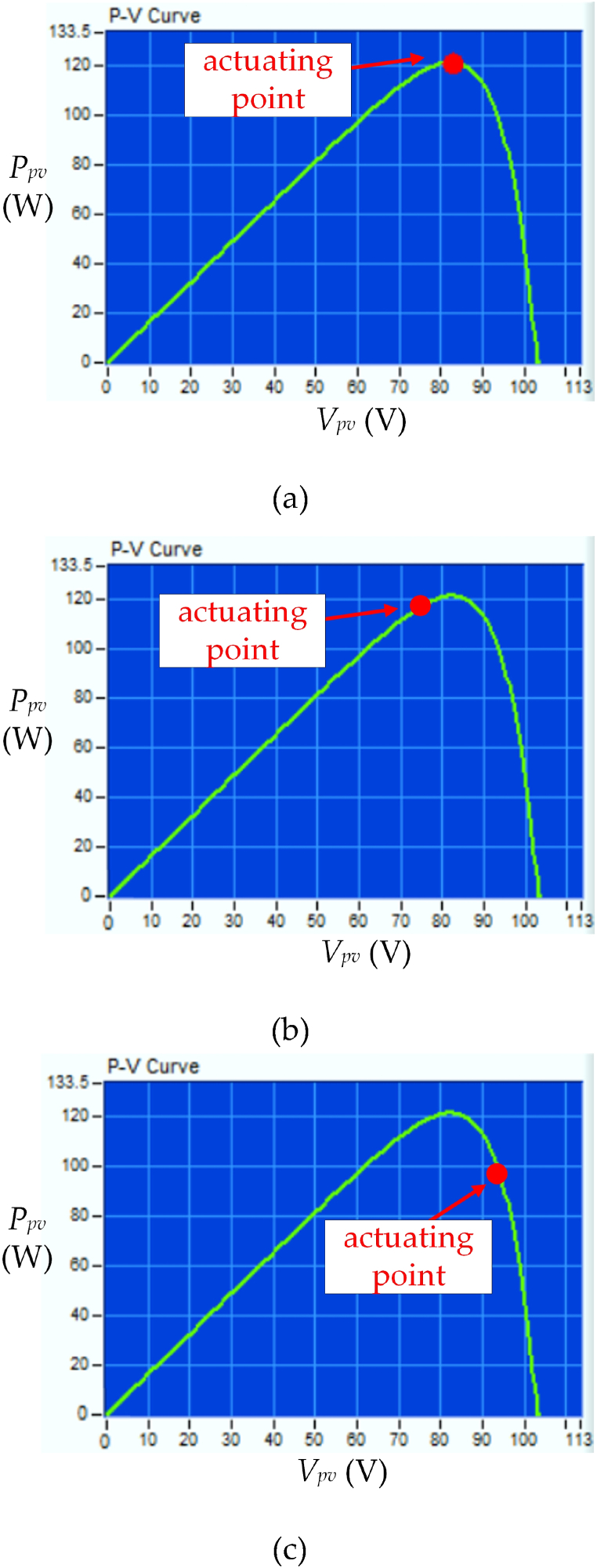
*P*_*pv*_−*V*_*pv*_ characteristic curve of three PV modules in series under UIC at irradiance level = 200 W/m^2^ and temperature = 25 °C by using different control strategies (a) HBD, (b) OPLR, and (c) P&O algorithms.

Fig. 11 shows the *P*_*pv*_−*V*_*pv*_ characteristic of three PV modules in series under UIC at irradiance level = 200 W/m^2^ and temperature = 25 °C using different control strategies. Fig. 11(a) displays the obtained MPP by using the proposed HBD algorithm which is *P*_*MPP*_ = 120 W and an efficiency of 99 %(as Fig. 10 (a)). Fig. 11(b) shows the actuating points using the OPLR algorithm *P*_*MPP*_ is 118 W and efficiency of 97 %. That is because this algorithm has mild operation to capture the MPP. Fig. 11(c) shows the actuating points using the P&O algorithm *P*_*MPP*_ is 94.5 W and efficiency of 78 %. This is because the P&O algorithm does not work well in low irradiance levels and the perturbation characteristics cause lower system efficiency.

Table 3 shows the efficiency comparison of HBD, OPLR, and P&O algorithms under UIC. The efficiency of the proposed HBD algorithm is 99 % under irradiance levels of 850 W/m^2^ and 200 W/m^2^, respectively. The efficiency of proposed HBD algorithm is higher than the OPLR, and P&O algorithms.

### 4.2 Experimental results of PSC

**Fig. 12:**
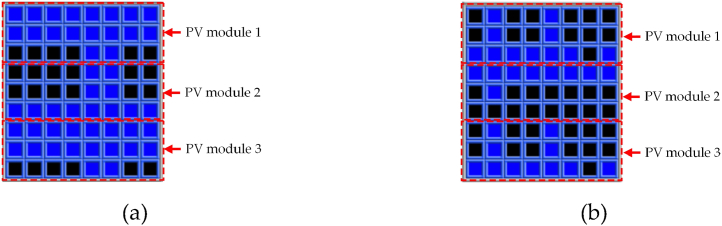
Schematic diagram of the three PV modules connected in series while performing shadow simulation in the solar photovoltaic simulator at (a) irradiance level = 600 W/m^2^ and temperature = 25 °C; and (b) irradiance level = 100 W/m^2^ and temperature = 25 °C.

Fig. 12 is the schematic diagram of the three PV modules connected in series performing the shadow simulation in a solar photovoltaic simulator. Fig. 12(a) displays three series-connected PV modules having 72 solar cells, irradiance level = 600 W/m^2^ and temperature = 25 °C; and 24 of them were shaded. At this point, PV module *V*_*oc*_ = 109 V, *I*_*sc*_ = 4.805 A, *V*_*MPP*_ = 50.7 V, *I*_*MPP*_ = 4.334 A, and *P*_*MPP*_ = 220 W. Fig. 12 (b) displays three series-connected PV modules having 72 solar cells, irradiance level = 100 W/m^2^ and temperature = 25 °C; and 41 of them were shaded. At this point, PV module *V*_*oc*_ = 98.4 V, *I*_*sc*_ = 0.789 A, *V*_*MPP*_ = 30.2 V, *I*_*MPP*_ = 0.625 A, and *P*_*MPP*_ = 18.9 W.

**Fig. 13:**
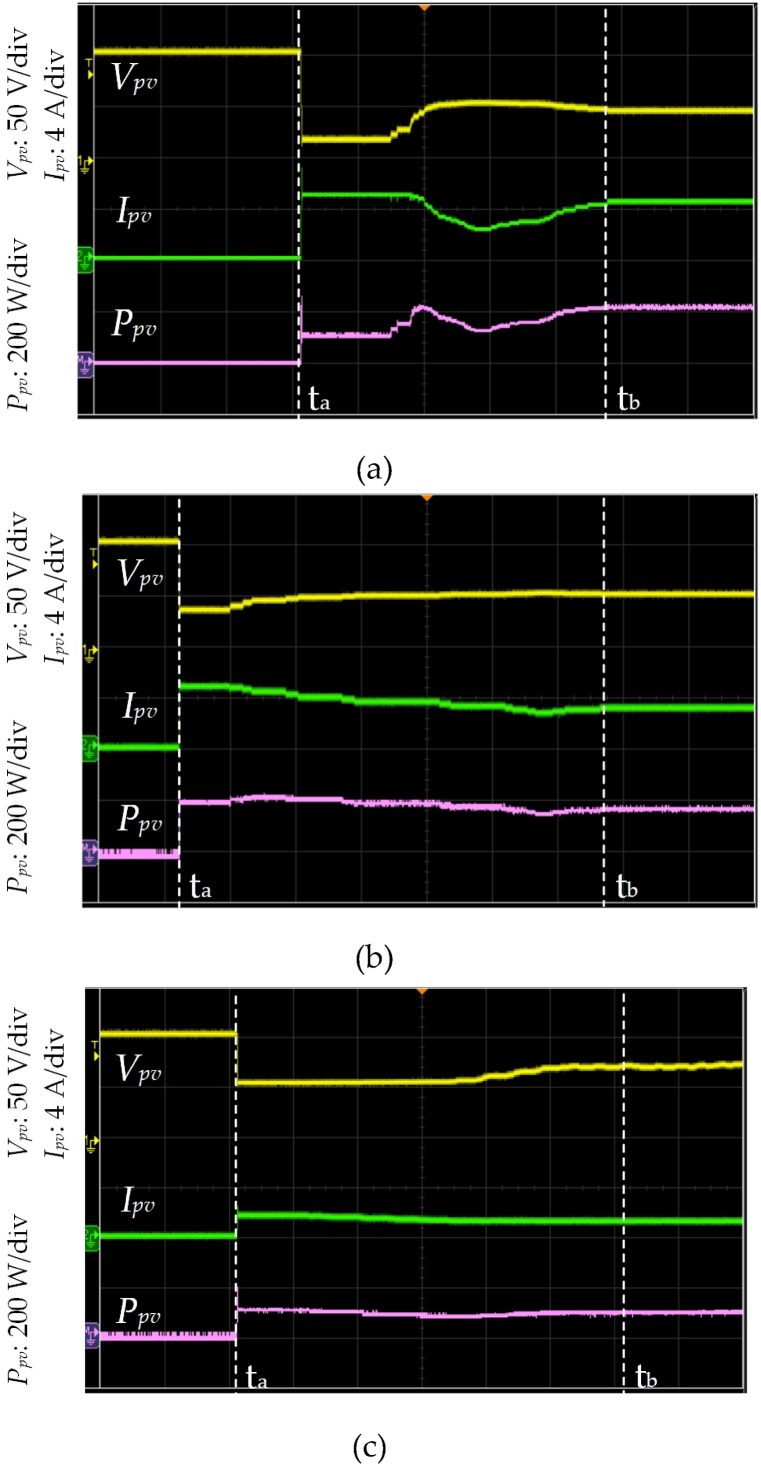
The waveforms of *V*_*pv*_, *I*_*pv*_, and *P*_*pv*_ under PSC with irradiance level = 600 W/m^2^ and temperature = 25 °C by using different control strategies (a) HBD, (b) OPLR, and (c) P&O algorithms (Horizontal axis: 1 ms/div).

**Fig. 14:**
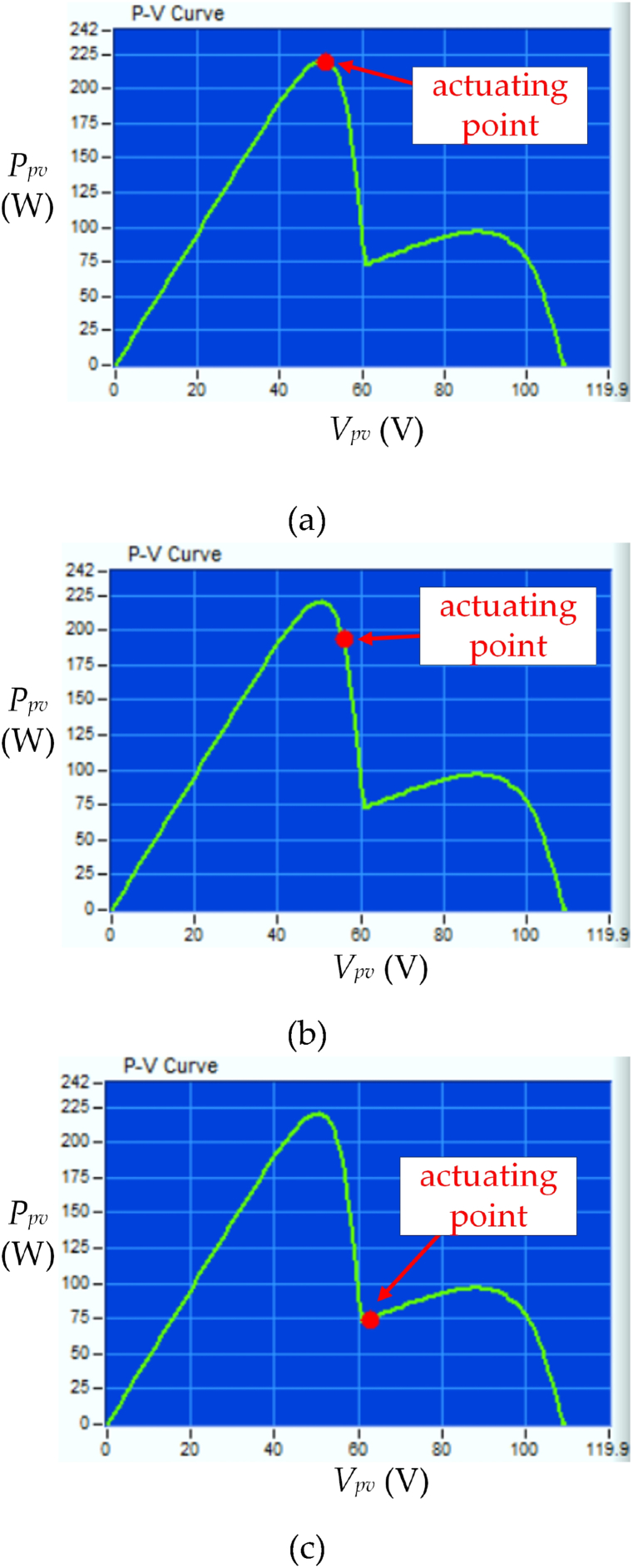
*P*_*pv*_−*V*_*pv*_ characteristic curve of three PV modules in series under PSC with two peaks at irradiance level = 600 W/m^2^ and temperature = 25 °C by using different control strategies (a) HBD, (b) OPLR, and (c) P&O algorithms.

Fig. 13 shows the waveforms of *V*_*pv*_, *I*_*pv*_, and *P*_*pv*_ under PSC with irradiance level = 600 W/m^2^ and temperature = 25 °C by using different control strategies. Fig. 13 (a) displays the proposed HBD algorithm. The MPPT begins at time t_a_ when *I*_*pv*_ > 0 is detected. The *P*_*pv*_−*V*_*pv*_ characteristic is analyzed using five honey bees which correspond to duty cycles of 0.05, 0.25, 0.5, 0.75, and 0.95 and resulting in respective *P*_*pv*_ of 25 W, 125 W, 205 W, 90 W, and 30 W. The slope is too steep between these five power points. So, the three PV modules in series under PSC are further verified using the proposed algorithm. Secondly, the maximum peak power is 205 W, the left power point is 125 W, and the right power point is 90 W. The proposed algorithm evaluates the MPP interval is between the left power point (125 W) and maximum peak power point (205 W), and *D*_1*st*_ = 0.25, *D*_*B1*_ = 0.31, *D*_*B2*_ = 0.38, *D*_*B3*_ = 0.44, and *D*_2*nd*_ = 0.5 are obtained through Eqs. (1), (2), (3), (4) (as shown in Fig. 5, Fig. 6). *P*_*pv*_ of 125 W, 150 W, 200 W, 218 W, and 205 W are obtained corresponding to *D*_1*st*_, *D*_*B1*_, *D*_*B2*_, *D*_*B3*_, and *D*_2*nd*_ respectively. The proposed HBD algorithm uses the iterative computation to capture the MPP at time t_b_ and the achieving MPPT time of 4.7 ms. The actuating point using the proposed HBD algorithm is *P*_*MPP*_ = 218 W, an efficiency of 99 %, and an MPP duty cycle of 0.44 (as shown in Fig. 14(a)). The proposed HBD algorithm can accommodate a large *V*_*pv*_ variation range while accurately identifying the MPP position.

Fig. 13 (b) shows the OPLR algorithm and the MPPT was started at time t_a_. This algorithm exhibits a gradual approach, and at time t_b_, the actuating point was *P*_*pv*_ = 189.2 W. However, performance of algorithm does not show further improvement, with the time elapsed from t_a_ to t_b_ being 6.5 ms (as shown in Fig. 14(b)). Fig. 13 (c) shows the P&O algorithm initiating MPPT at time time t_a_ and reaching an actuating point of *P*_*pv*_ = 74.8 W at time t_b,_ with a time span of 6 ms (as shown in Fig. 14(c)). The P&O algorithm led *V*_*pv*_ keeps oscillating and it is not suitable for operation under PSC, so the system performance is low.

Fig. 14 shows the *P*_*pv*_−*V*_*pv*_ characteristic curve of three PV modules in series under PSC with two peaks at irradiance level = 600 W/m^2^ and temperature = 25 °C using different control strategies. Fig. 14 (a) displays the *P*_*pv*_−*V*_*pv*_ curve using the proposed HBD algorithm. The actuating point using the proposed HBD algorithm is *P*_*MPP*_ = 218 W, an efficiency of 99 % (as shown in Fig. 13(a)). Fig. 14(b) and (c) show that the actuating points of OPLR and P&O algorithms were 189.2 W and 74.8 W and the efficiency of 86 % and 34 %, respectively (as depicted in Fig. 13(b) and (c)).

**Fig. 15:**
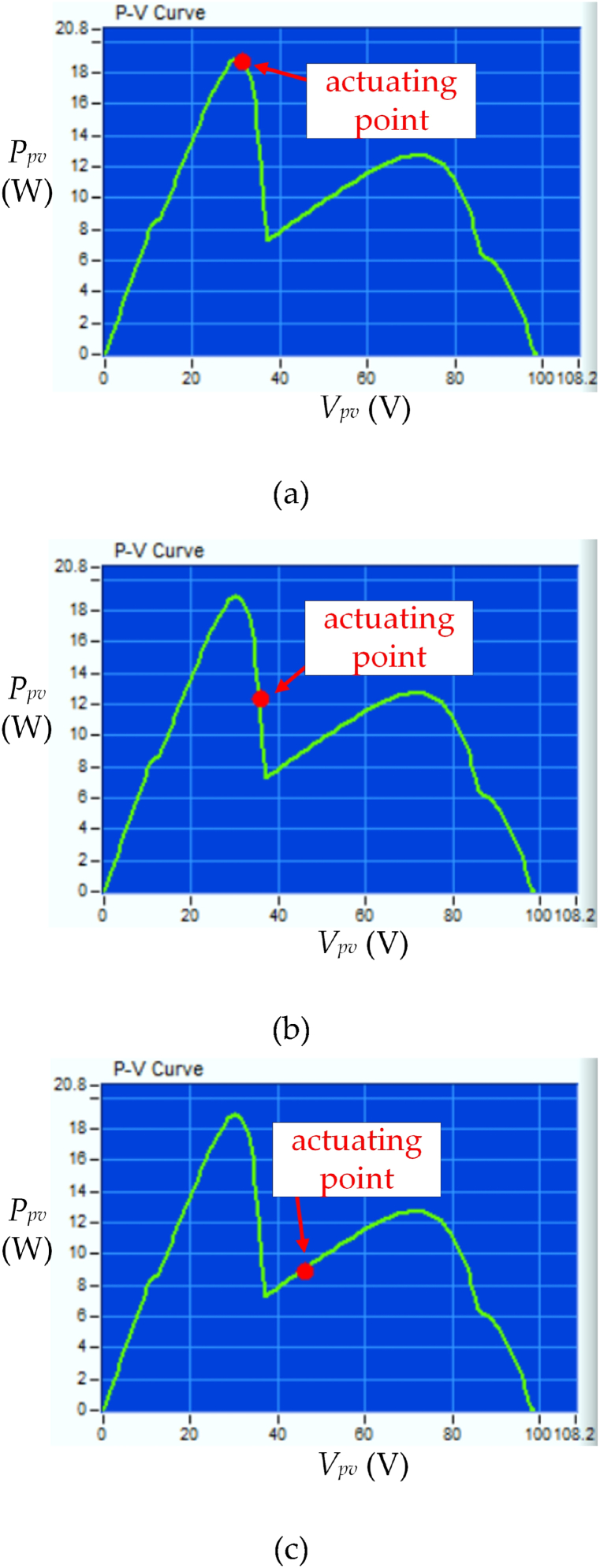
*P*_*pv*_−*V*_*pv*_ characteristic curve of three PV modules in series under PSC with three peaks at irradiance level = 100 W/m^2^ and temperature = 25 °C by using different control strategies (a) HBD, (b) OPLR, and (c) P&O algorithms.

Fig. 15 shows the *P*_*pv*_−*V*_*pv*_ characteristic of three PV modules in series under PSC with three peaks at irradiance level = 100 W/m^2^ and temperature = 25 °C using different control strategies. Fig. 15(a) displays the obtained MPP by using the proposed HBD algorithm which is *P*_*MPP*_ = 18.7 W and an efficiency of 99 %. Fig. 15(b) and (c) show that the actuating points using the OPLR and P&O algorithms which are 12.3 W and 8.8 W and the efficiency of 65 % and 47 %, respectively. This is because of the challenge faced by conventional MPPT algorithm in reaching MPP under low irradiance levels and PSC.

Table 4 shows the efficiency comparison of HBD, OPLR, and P&O algorithms under PSC. The efficiency of proposed HBD algorithm is 99 % under irradiance levels of 600 W/m^2^ and 100 W/m^2^, respectively. The efficiency of proposed HBD algorithm is higher than the OPLR, and P&O algorithms.

Table 5 shows the comparison of HBD, OPLR, and P&O algorithms’ convergence time. The convergence time of the proposed HBD algorithm under UIC with an irradiance level of 850 W/m^2^ is 5.1 ms while under PSC with an irradiance level of 600 W/m^2^, the convergence time is 4.7 ms. The convergence time of the proposed HBD algorithm is better than the OPLR, and P&O algorithms.

**Table 5:** Comparison of convergence time of HBD, OPLR, and P&O algorithms.

Algorithm	Convergence time	
	**Irradiance level of 850 W/m**^**2**^**under UIC**	**Irradiance level of 600 W/m**^**2**^**under PSC**
OPLR	5.8 ms	6.5 ms
P&O	7.2 ms	6 ms
HBD	5.1 ms	4.7 ms

## 5 Conclusion

This study proposes a novel HBD MPPT algorithm. This control strategy is inspired from the remarkable behavior of bees. When a bee finds nectar, it returns to the honeycomb and dances to communicates the nectar location with other bees. Other bees then fly towards the location and gather the nectar (MPP position). This method combined the nectar-gathering behavior of bees with solar power generation to optimize the solar power generation.

Firstly, the efficiency of proposed HBD algorithm is 99 % at irradiance levels of 850 W/m^2^ and 200 W/m^2^ under UIC. The OPLR algorithm yields an efficiency of 99% and 97% respectively, while the P&O algorithm lags at 99% and 78% efficiency. The proposed algorithm performed better than the OPLR and P&O algorithms. Secondly, the efficiency of proposed algorithm is 99 % at irradiance levels of 600 W/m^2^ and 100 W/m^2^ under PSC. The efficiency of the OPLR algorithm were 86 % and 65 % respectively while these values were respectively 34 % and 47 % for the P&O algorithm. In this case also, the performance of HBD algorithm is higher than the OPLR and P&O algorithms. Thirdly, the proposed HBD algorithm outperformed the OPLR, and P&O algorithms while examining convergence times. It achieves a remarkable convergence time of 5.1 ms under UIC with an irradiance level of 850 W/m^2^ and an even faster convergence time of 4.7 ms under PSC with an irradiance level of 600 W/m^2^. The proposed HBD algorithm consistently demonstrates a higher efficiency than both the OPLR algorithm and P&O algorithm. This method is free of PV module specification data, complex calculations, and additional temperature meters and heliographs. It is highly efficient and has significant economic benefits.

Future developments are warranted to minimize the time for bees to search the MPP and thus enhancing its overall performance. Since, the control strategy based on this method is expected to obtain optimal system effectiveness, the proposed HBD algorithm can be extended into large-scale PV array systems for MPPT in the future. In addition, the proposed HBD algorithm can also be used in hybrid PV generation systems. When the irradiance level is insufficient for the PV module to produce an output to meet the load demand, the energy storage battery will supply the electricity to the load. The proposed HBD algorithm can be seamlessly integrated into such systems and effectively combining the limited electricity generated by the PV module with the energy stored in batteries to enhance system effectiveness in supplying electricity for the load.

## Funding statement

This research was funded by the 10.13039/100020595National Science and Technology Council, Taiwan, grant number MOST 112-2221-E−003-003, the NTUS innovation cooperation 11312111001, and the 10.13039/501100006397National Taiwan Normal University (NTNU), Taiwan.

## Data availability statement

Data will be made available on request.

## CRediT authorship contribution statement

**Wei-Jen Chen:** Funding acquisition, Writing – original draft, Writing – review & editing. **Shoeb-Azam Farooqui:** Writing – original draft, Writing – review & editing. **Hwa-Dong Liu:** Conceptualization, Data curation, Formal analysis, Funding acquisition, Methodology, Supervision, Writing – original draft, Writing – review & editing. **Shan-Xun Lai:** Writing – original draft. **Ping-Jui Lin:** Writing – original draft.

## Declaration of competing interest

The authors declare that they have no known competing financial interests or personal relationships that could have appeared to influence the work reported in this paper.
